# Linear and Nonlinear Schemes Applied to Pitch Control of Wind Turbines

**DOI:** 10.1155/2014/406382

**Published:** 2014-09-03

**Authors:** Hua Geng, Geng Yang

**Affiliations:** Department of Automation, Tsinghua University, Beijing 100084, China

## Abstract

Linear controllers have been employed in industrial applications for many years, but sometimes they are noneffective on the system with nonlinear characteristics. This paper discusses the structure, performance, implementation cost, advantages, and disadvantages of different linear and nonlinear schemes applied to the pitch control of the wind energy conversion systems (WECSs). The linear controller has the simplest structure and is easily understood by the engineers and thus is widely accepted by the industry. In contrast, nonlinear schemes are more complicated, but they can provide better performance. Although nonlinear algorithms can be implemented in a powerful digital processor nowadays, they need time to be accepted by the industry and their reliability needs to be verified in the commercial products. More information about the system nonlinear feature is helpful to simplify the controller design. However, nonlinear schemes independent of the system model are more robust to the uncertainties or deviations of the system parameters.

## 1. Introduction

Wind power generation has widely grown during the last decades and nowadays is the most competitive form of renewable energy [1].

**Figure 1:**
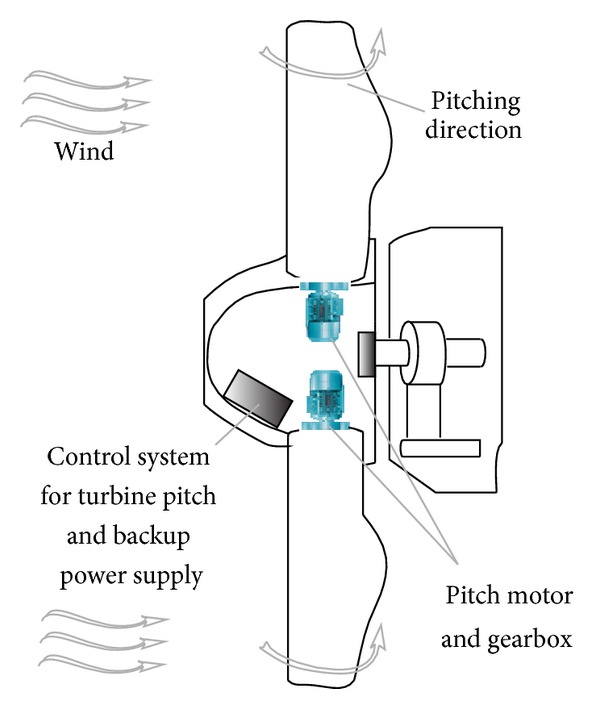
Configuration of pitch regulation subsystem in the WT.

Wind turbines (WTs) are usually designed to withstand extreme winds statically so that they can survive a storm. However, such property keeps true only when the turbine is not spinning. At very large rotational speeds, the forces on the blades and other parts of the turbine are enormous and they will literally tear the turbine apart. In order to deal with the mechanical stress posed to the turbines during high wind speed, pitch-regulated and stall-regulated strategies are proposed and have been employed in the commercial WTs [2]. Pitch-regulated WECS have an active control system to vary the pitch angle (turn the blade around its axis) as shown in Figure 1. The torque produced by the turbine blades and the rotational speed are, thus, decreased. Stall-regulated WECS, on the other hand, have the blades specially designed so that the rotational speed and the aerodynamic torque decrease with increasing wind speed above a certain value.

**Figure 2:**
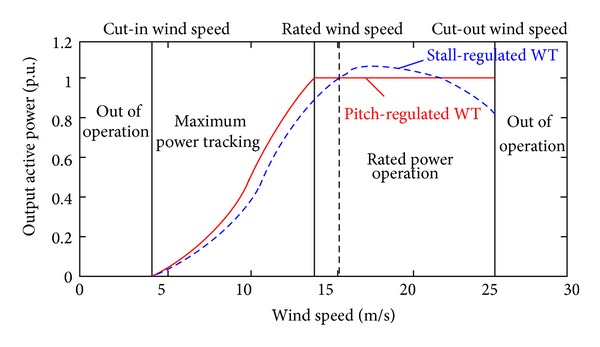
Power curves of fixed pitch and variable pitch wind turbines.

The typical power curves of the pitch and the stall-regulated WECS are shown in Figure 2 [2, 3]. Both pitch and stall regulations are activated when wind speed is high [2, 4]. Although the stall regulation requires less moving parts and capital cost, the pitch regulation is more flexible and results in higher power quality and efficiency and lower stress on the mechanical parts. Therefore, it is more widely applied in the commercial WTs.

As analyzed in [1], the control of the pitch angle is not easy because the system behavior is highly nonlinear. Many studies have been reported about the WT pitch control [1–14]. Generally, the proposed schemes can be classified into two categories, linear based and nonlinear based methods. Linear controller is designed based on linear system model. If the system behavior is highly nonlinear, linearization of the system model around a certain operation point is usually necessary. Then, linear controller can be designed on the basis of the small-signal linear model [4, 5]. Following the above procedures, the system performance can be promised around the linearization point. When the system operation point deviates from the linearization one, the performance is deteriorated [1]. Gain scheduling linear controller was proposed to improve the control performance in the whole operation range [4, 6–8]. In this method, a family of linear dynamic controllers with different gains is designed for a family of plant linearization about constant operating points. In order to determine the gains, plenty of simulations or even experiments are required [4]. Nonlinear strategies are another alternative choice for the WT pitch control, which can be designed based on both linear and nonlinear system models [1, 11–14]. As illustrated in previous studies, nonlinear schemes are prone to providing better response, such as rapid pitch tracking, low regulating overshoot, and low mechanical stress, compared with linear schemes. However, most of the studies consider little about the real nonlinear property of the WT. Moreover, such strategies are usually complex to be implemented and difficult to be understood by the field engineers.

This paper analyzed the nonlinear property of the real WT and compared the typical linear and nonlinear strategies employed in the WT pitch control from the engineering point of view. The procedure of parameter design for each strategy is introduced and the final controller is applied in the digital simulations. The performance and engineering practice of different strategies are then compared with the simulation results.

## 2. System Configuration and Modeling

**Figure 3:**
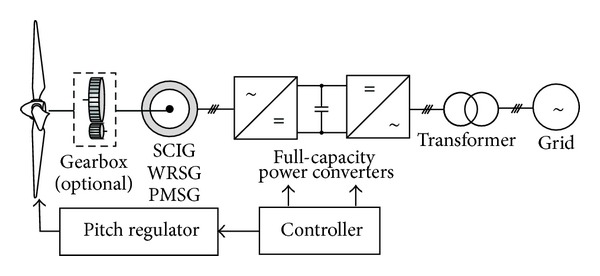
Configuration of a typical WECS (with full-capacity power converters).

The configuration of a typical WECS is shown in Figure 3, which is composed of three subsystems: an electrical subsystem composed of the generator and the chopper-inverter equipment, the pitch angle subsystem composed of the pitch angle controller and its actuator, and the turbine mechanical subsystem [1].

**Figure 4:**
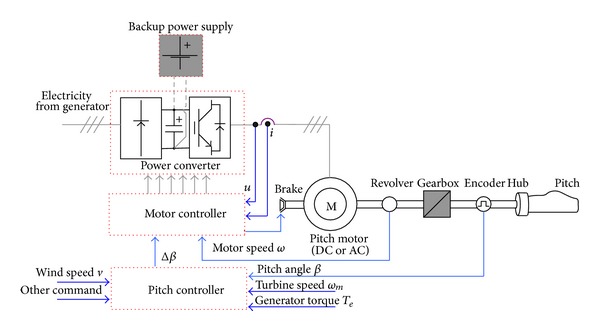
Configuration of pitch regulation control system in the WECS.

Usually, pitch system can be hydraulic or electrical. The electrical pitch system uses the converter driven servomotor to regulate the pitch angle, which provides simpler structure, smaller size, and more convenient maintenance compared with the hydraulic one. The typical configuration of the electrical pitch system is illustrated in Figure 4. The pitch motor is driven with a power converter with the electricity powered by the generator or the backup power supply in case of low wind speed or system fault. Pitch controller can respond to the command from the high-level main controller. On the other hand, it determines the pitch angle biases with the linear or nonlinear control strategies based on the information of wind speed, turbine speed, generator torque, and so forth. After the calculation, pitch controller generates the pitch angle command and sends it to the motor controller. The motor controller samples the motor speed and the converter voltage and current. And then it produces the PWM pulses to drive the power converter. With the closed loop control, the pitch angle would track its command within several seconds. Due to the physical limitation of the servosystem, the typical maximal pitch regulation rate is ±10°/s. Moreover, a “dead zone” is necessary to ignore commanded pitch rates less than ±0.1°/s to reduce the actuator motion and eliminate noise in the command signal.

Compared with the pitch system, other subsystems have different response time scales. For example, the electrical system of the power generation responds quickly (in milliseconds) due to the small time constant determined by the stator and rotor impedance. In contrast, the turbine speed varies slowly (in multiminutes) because of the large inertia. Therefore, the pitch regulator is slower than the electrical response but much rapider than the mechanical response.

Considering the time-scale difference between different subsystems, the system modeling can be simplified. Specifically, the electrical dynamic can be ignored in the evaluation of pitch and mechanical dynamics. And the pitch actuator dynamic can be simply represented by a first-order model [1].

Based on the above assumption, the mathematical model of the pitch-regulated WECS can be expressed as

(1) ω˙m=1JTωt−Te′,Tactβ˙+β=βc,

where *ω*_*m*_ is the rotational turbine speed. *T*_*ωt*_ is the mechanical torque provided by a wind turbine. *T*_*e*_′ = *K*_*g*_ *T*_*e*_ is the equivalent generator torque, *K*_*g*_ is the ratio of gearbox, and *T*_*e*_ is the electromagnetic torque of the generator. *J* = *K*_*g*_^2^ *J*_*g*_ + *J*_*ωt*_ is the equivalent turbine inertia, *J*_*g*_ is the generator moment of inertia, and *J*_*ωt*_ is the wind turbine moment of inertia. *T*_act_ is the time constant of the pitch actuator and *β*_*c*_ is the command of the pitch angle.

In (1), *T*_*ωt*_ is described as

(2) $$\begin{array}{c} T_{\omega t}=\frac{1}{2}C_{q}\pi R^{3}\rho v^{2}, \end{array}$$

where *C*_*q*_ = *f*(*λ*, *β*) is the torque coefficient. *R* is the radius of the wind turbines. *ρ* is the air density. *v* is the wind speed and *λ* = *ω*_*m*_ *R*/*v* is the tip speed ratio.

**Figure 5:**
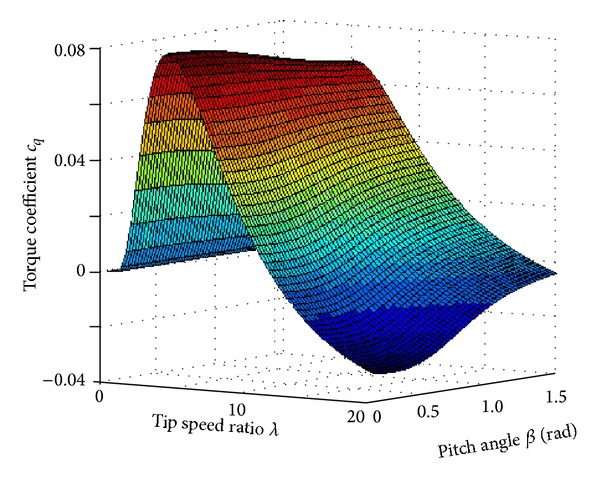
Typical torque coefficient curve.

The torque coefficient *C*_*q*_ is a highly nonlinear function of the tip speed ratio and the blade-pitch angle. The typical *C*_*q*_ curve can be illustrated in Figure 5.

The WT characteristic shown in Figure 5 can be approximated with first-order or high-order polynomials [15–17]. One example is as

(3) Cq=0.22116λi−0.4β−5e−12.5/λi,1λi=1λ+0.008β−0.035β3+1.

Equation (3) provides good approximation to the typical toque coefficient. In real applications, turbine manufactory should implement repetitive wind tunnel tests to obtain *C*_*q*_. Experiments at different wind speed and turbine rotational speed are repeated so that a discrete data table with the information at different steady-state operation points is obtained. First of all, different types of WT should have different characteristics. Therefore, approximation with unique polynomials or transcendental equation cannot represent the characteristics of WT. Usually, the turbine blades are manufactured locally. By establishing the turbine factory close to the spot of wind farm, the transportation costs for the turbine blades can be saved. In the temporary turbine factory, the consistency of the production process is difficult to guarantee. As a result, the WT characteristics with even the same type are different from one another. Besides the above phenomenon of real engineering practice, parameter uncertainties are also very common in the WTs. The WECS operates outdoors and the system should resist the erosion of wind sand, snow rainfall, salt spray, and other circumstance. Because of the environmental changes, the WT characteristics are varying which bring difficulties in the modeling and control design for the WECS. When considering the measurement error of the wind speed experienced by the turbine blades, the situation becomes more complicated.

The alternative choice to obtain the WT characteristics is to fit Figure 5 from the set of discrete experimental data supplied by the WT manufactory. It provides much more accurate description of the nonlinear aerodynamic characteristics although error still appears when considering the environmental variation. Because the data set is different from one turbine blade to another, control parameters adaptive to one turbine sometimes cannot be employed in another WT. Therefore, design and tuning of the control parameters are tedious, which is not good to the commercialization of the pitch system.

As indicated by the above analysis from the engineering point of view, it is concluded that the nonlinear property, the parameter uncertainty, and the implementation cost are the main difficulties of the pitch control.

## 3. Controller Design

Generally, the control scheme can be designed based on a linear or nonlinear system model. Taking account of the controller property, there are four controller design principles, linear controller based on linear system model (LCLM), nonlinear controller based on linear system model (NCLM), linear controller based on nonlinear system model (LCNM), and nonlinear controller based on nonlinear system model (NCNM). Since the real property of the WT aerodynamic characteristics is highly nonlinear, NCLM is not an efficient way to solve the pitch control problem. In the left three principles, LCLM is employed in early WECSs and the proportional-integral (PI) controller is normally used. The PI parameters are designed based on the linearized system model and try and error methods are necessary to obtain the satisfactory performance in the real pitch system. However, such strategy is proven to be noneffective when the operation point of the WECS deviates from the linearized working point [1]. In contrast, LCNM and NCNM are more adaptive to the control of such nonlinear systems. The design principle and their performance are analyzed as follows.

### 3.1. LCNM Based Scheme

LCNM is studied to avoid the performance deterioration of LCLM at different operation points [4]. The simplest way to achieve LCNM is to design different PI parameters based on a family of linearized system model at different operation points and schedule the gains when operation point deviates. The parameter uncertainty is tolerated by the linear controller at the same time.

In this method, several operation points at different wind speed are firstly selected, for example, 12 m/s, 15 m/s, 18 m/s, 21 m/s, and 25 m/s. It is noted that selection of the operating point is critical to ensure the aerodynamic stability and pitch regulation performance in the system. Principles in details can be found in [4]. Afterwards, the linearized system models are obtained at each working point. Taking one point as an example, assume

(4) $$\begin{array}{c} {\left.T_{\omega t}\right|}_{\text{OP}}={\left.T_{e}^{\prime }\right|}_{\text{OP}}, \end{array}$$

where subscript “OP” means the corresponding operating point. Because the electrical dynamic is quick enough, *T* ^′^_*e*_|_OP_ can be considered as a constant. Therefore, linearization of the turbine equation (1) results in the following model:

(5) $$\begin{array}{c} J\Delta {\dot{\omega }}_{m}=\gamma \Delta \omega_{m}+\alpha \Delta v+\zeta \Delta \beta , \end{array}$$

where the linearization coefficients are given by

(6) γJ∂ω˙m∂ωmOP=12ρπR4vOP∂Cq∂λOP,αJ∂ω˙m∂vOP=12ρπR3vOP2CqOP−λOP∂Cq∂λOP,ζJ∂ω˙m∂βOP=12ρπR3vOP2∂Cq∂βOP;

here, $\Delta {\dot{\omega }}_{m}$, Δ*ω*_*m*_, Δ*v*, and Δ*β* represent the deviations of ${\dot{\omega }}_{m,\text{OP}}$, *ω*_*m*,OP_, *v*_OP_, and *β*_OP_, respectively.

Based on the linear model described by (5), zero-pole assignment method in linear control theory can be adopted to design the PI parameters.

**Figure 6:**
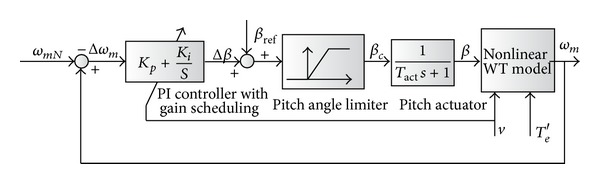
System frame of LCNM based scheme.

The frame of the gain scheduled LCNM is illustrated in Figure 6. Once the turbine speed exceeds its rating, the error will command a change in blade-pitch angle (Δ*β*) which results in the cut-down of wind energy absorption. The pitch angle *β*_*c*_ = Δ*β* + *β*_ref_ is physically limited to angles between 0° and 60°. The reference pitch angle *β*_ref_ can remain its optimal value. The PI parameters are changed with wind speed or system operation point.

With gain scheduling, the controller can provide satisfactory performance. However, the implementation cost of the scheme is great. Plenty of simulations or even experiment tests should be executed to get the PI parameters at different working points [4].

### 3.2. NCNM Based Scheme

In contrast to LCNM, NCNM based scheme seems more effective in the inherent nonlinear WECS. Generally, NCNM can be classified into two categories. One is the scheme depending on the system model and the other is the one independent of the nonlinear model.

In [17], the WT characteristics are approximated by the nonlinear high-order polynomials and the pitch controller has the similar nonlinear structure. Such design can result in good performance, but the approximated system model should be known in advance. For different WTs, the parameters in the polynomials should be estimated and the controller design procedure is repeated. Moreover, the uncertainties in the system model are not considered in the method, which can cause control errors in real applications.

**Figure 7:**
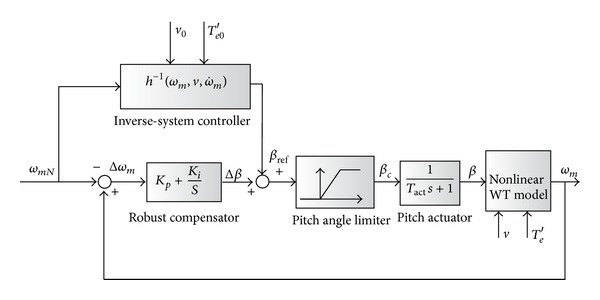
System frame of model dependent NCNM scheme.

Reference [1] also proposed a nonlinear scheme based on the WT model. Being different from [17], the system model is fitted from the set of discrete experimental data supplied by the WT manufactory. Its advantage is that the nonlinear controller utilizes the characteristics data directly and thus can provide better performance than that of a linear controller. For different types of WT, the experimental data is changed and inputted into the controller while the structure of the controller is identical. Moreover, the parameter uncertainty can be tolerated by additional robust control loop in [1]. The frame of this model based NCNM is shown in Figure 7, where *v*_0_ is the measured wind speed, *T*_*e*0_′ is the real equivalent generator torque, and $h^{-1}\left(\omega_{m},v,{\dot{\omega }}_{m}\right)$ is the inverse-system of the WT model.

In Figure 7, the inverse-system can be calculated with (1) and (2) by considering *ω*_*m*_, *v*, *T*_*ωt*_, *T*_*e*_′, and the WT characteristics as the known parameters and *β* as the unknown variable. To implement the scheme, WT characteristics are represented by the experimental data from the WT manufactory. *ω*_*m*_, *v* are detected by sensors. The output of the inverse-system controller produces a pitch angle reference *β*_ref_. By doing this, the nonlinear feature of the WT characteristics can be counteracted and the tracking error due to the system nonlinear property is very small. In real applications, both the wind speed measurement and generator torque control can have control errors. The parameter uncertainty also emerges with the daily operation of the WECS. All the above reasons can affect the accuracy of the pitch control. To avoid those facts, a robust compensator with the PI feature is included in Figure 7. Since the inverse-system controller achieves the dominant pitch regulation, the robust compensator produces only small pitch angle deviation Δ*β* and its parameters are easy to tune without gain scheduling.

**Figure 8:**
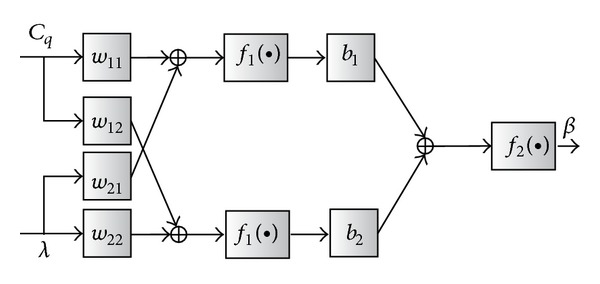
Topology of the 3-level BP neural network.

In contrast to model dependent NCNM scheme, model independent NCNM strategy employs adaptive nonlinear controller to fit the system nonlinear feature especially when the operation point drifts. Artificial neural network (ANN) is a good choice to achieve the control objective. In [12], a 3-level back propagation (BP) neural network is adopted in the pitch control. The topology of the BP network is denoted in Figure 8, in which *w*_*ij*_, *b*_*i*_  (*i*, *j* = 1,2) are the network weights and *f*_1_(•) and *f*_2_(•) are the transfer functions of the BP network. Here, *f*_1_(•) and *f*_2_(•) are chosen to be the Tan-Sigmoid and Log-Sigmoid models, respectively.

The discrete aerodynamic experimental data from the turbine manufactory is used as the training data for the neural network. As shown in Figure 8, the neural network has two inputs, *λ* and *C*_*q*_, and one output *β*.

**Figure 9:**
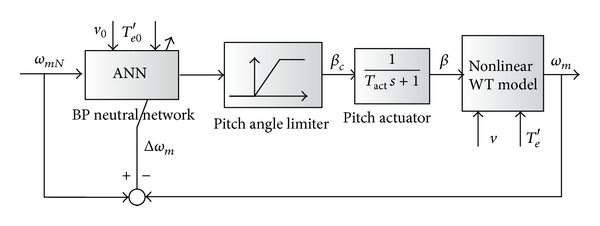
System frame of model independent NCNM scheme.

Basically, the ANN controller utilizes the neuron to identify the nonlinear system model or inverse model and applies the identification results into the control.  Figure 9 is an example of the ANN controller for pitch control (or called neural network inverse control). In the frame, ANN with topology of Figure 8 is employed, which provides the inverse model of the WT characteristics. In order to avoid the control errors and instability due to the open loop control structure, the turbine speed error is fed back to the ANN to change the network weight online. By doing this, the wind speed measurement error and control error of the generator torque can also be tolerated.

As the nonlinear feature is identified in the controller, it can show a convincing performance as shown in [12]. However, the training process of the neural network and the online update of the network are complicated, which requires a powerful digital processor and takes time.

## 4. Simulation Results

Simulations are carried out using MATLAB/SIMULINK to compare the performance of three above different pitch controllers (with frame shown in Figures 6, 7, and 9). The nonlinear aerodynamic characteristics of WT in the simulation models are provided by the RISØ National Laboratory of Denmark, and the data considers the blade-element momentum analyses and empirical models that predict stalled operation and blade tip losses. The nominal parameters of the WECS in the simulations are listed in Table 2.

### 4.1. Performance with the Operation Points Variation

**Figure 10:**
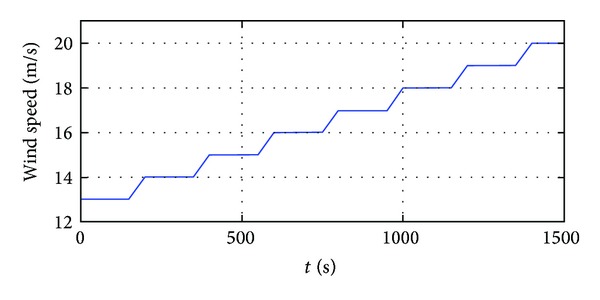
Input wind speed.

**Figure 11:**
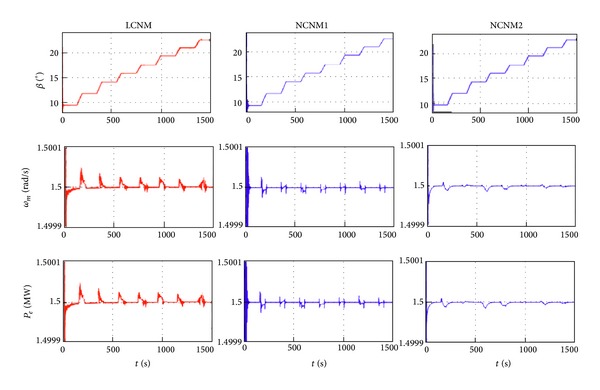
System responses with different pitch controllers in the step wind situation.

The steady-state operation points are varied with the change of wind speed. In the simulation, the wind speed (above the rated value) is step changing with the profile in Figure 10. System responses with LCNM and two NCNM controllers are illustrated in Figure 11.

As indicated by Figure 11, three controllers all work well in the entire wind speed region. The turbine speed and output power of the WECS can remain its nominal value even with the wind speed step. The details of the system response with LCNM controller seem worse than those of the NCNM controllers. It is reasonable because only the information of several working points is used in the controller design for the LCNM scheme while the NCNM strategies consider the system nonlinear features within all the operation regions. Theoretically, two NCNM strategies should have similar responses, but it is not the case shown in Figure 11. The reason is that the model based NCNM scheme (inverse-system controller in [1]) utilizes the linear interpolation method to generate the continuous inverse-system from the discrete aerodynamics data while the system model adopts nonlinear interpolation method in the simulations. In the model independent NCNM scheme, the ANN can track the nonlinear feature of the system model and, therefore, can provide better response. However, it is deduced that the performance of the model based NCNM can be close to that of the ANN controller if more discrete data of the WT characteristics are available. Such requirements increase the burden on the experimental tests of the WT manufactories.

### 4.2. Performance with the Parameter Uncertainty

Parameter uncertainties are very common in the WECS which should be evaluated in the controller design. Uncertainties can come from the measurement and control errors or parameter deviations. In this section, simulation with measurement error of the wind speed is implemented. On the one hand, such error is inevitable in the real applications. On the other hand, control errors because of other parameter uncertainties can always be considered as the equivalent deviation on the wind speed. Moreover, from the simulation point of view, such scenario is easier to implement.

**Figure 12:**
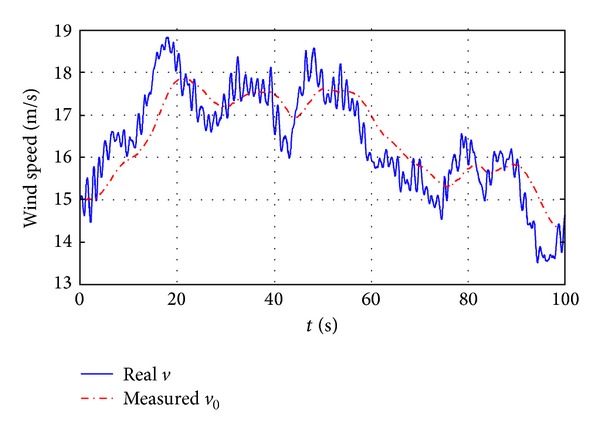
Real and measured wind speed.

**Figure 13:**
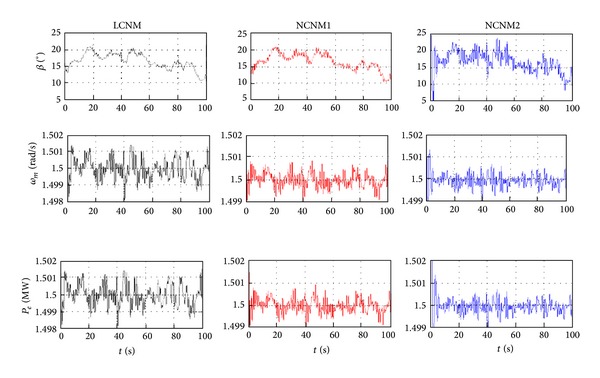
System responses with different pitch controllers considering parameter uncertainties.

In the simulation, it is assumed that the real wind speed *v* is different from the measured one *v*_0_ with the profile shown in Figure 12. The wind speed model is developed at RISØ National Laboratory based on the Kaimal spectra taking the tower shadow and the rotational turbulences into account. System responses with LCNM and two NCNM controllers are illustrated in Figure 13.

Figure 13 shows that all three schemes can withstand parameter uncertainties thanks to the close-loop control structure. The LCNM method results in larger control errors compared with the NCNM strategies because the PI gains are switched based on the detected wind speed. The ANN controller achieves the most accurate control of the turbine speed and output power as the neural network can track the system dynamic online even when the parameter is changing. However, smaller tracking errors would cost more frequent pitch action, which may increase the mechanical fatigue of the WT.

## 5. Discussion

As compared by the above analysis and simulation results, different control structure for system with nonlinear dynamics, such as the WECS, has its advantages and disadvantages. The comparisons are indicated in Table 1.

Considering the control performance, nonlinear scheme taking account of the system nonlinear feature provides smaller control errors compared with linear schemes. In all the nonlinear schemes, the one independent of system models has better performance because it can adaptively vary the control parameters on the basis of the system response. In contrast, nonlinear schemes based on system models cannot work well if the model parameters drift. The robust compensations, such as in Figure 7, are an effective way to improve its performance and are always necessary in the real applications.

From the engineering point of view, linear scheme with gain scheduling is attractive to the commercial products. Most of the time, the control performance is acceptable. More importantly, for the field engineers, it is easy to understand the principle of the scheme; therefore, the cost of field service and technique training is saved. Also, such scheme has already been applied in the products for many years and the reliability is widely accepted by the industry and consumers, which is a benefit to the business marketing. As for the cost, such scheme requires more efforts on the design of control parameters. Usually, try and error methods are used and plenty of simulations or experiments are necessary. If the consistency of the product parameters can be guaranteed, such scheme is a good choice. In contrast to linear scheme, the NCNM schemes increase the controller complexity. Although powerful digital processor can easily execute the nonlinear scheme nowadays, the reliability of such scheme needs time to be accepted by the industry.

## 6. Conclusion

This paper discusses the controller design of the pitch control for WTs from both the theoretical and the engineering point of view. Because of the highly nonlinear WT aerodynamics, linear controller with unique parameters cannot provide satisfactory performance. Sometimes, it results even in instability. Linear controller with gain scheduled parameter is a good choice from both implementation cost and industrial acceptance perspectives. If the better performance is required, nonlinear schemes are necessary. Essentially, the nonlinear strategies improve the control performance by employing the system nonlinear features. Strategies adaptive to the variation of system parameters or structures are deserved to get better results while the ones depending on the system models result in control errors when the model deviates. Selection of the control scheme is a comprehensive job affected by the ongoing techniques, performance, costs in terms of implementation, training, maintenance, and so forth.

## Figures and Tables

**Table 1:** Comparisons of different control schemes.

Schemes	Comparison			
	Control performance		Practical application	
	Steady-state error	Robustness	Complexity	Industrial acceptance
LCNM	Large	Good	Low	Good
NCNM1	Small	Better (with robust compensation) Bad (without robust compensation)	High	Bad
NCNM2	Smallest	Best	Highest	Worst

**Table 2:** Nominal Parameters of the WECS.

Parameters	Value
Blade radius [m]	40
Air density [kg*·*m^3^]	1.25
Cut in wind speed [m/s]	3
Cut out wind speed [m/s]	25
Rated wind speed [m/s]	12.6
Turbine Inertia [kg*·*m^2^]	90 × 10^6^
Rated Turbine Speed [rad/s]	1.5
Generator Inertia [kg*·*m^2^]	90
Gearbox Ratio	100
Generator Rated Torque [N*·*m]	1 × 10^6^
Generator Rated Power [MW]	1.5
Response time of the pitch actuator [s]	0.5
